# Supplementary material for: Light-Up Split Broccoli Aptamer as a Versatile Tool for RNA Assembly Monitoring in Cell-Free TX-TL Systems, Hybrid RNA/DNA Origami Tagging and DNA Biosensing
Source: Int J Mol Sci. 2023 May 9;24(10):8483. doi: 10.3390/ijms24108483 (PMC10218337; doi:10.3390/ijms24108483)
Supplement: Supplementary file 1 [file ijms-24-08483-s001.zip › ijms-2252382-supplementary.pdf]

SUPPLEMENTARY MATERIALS

Light-up split Broccoli aptamer as a versatile tool for RNA assembly monitoring in cell-free TX-TL systems, hybrid RNA/DNA origami tagging and DNA biosensing

Emanuela Torelli[1, *], Ben Shirt-Ediss[1], Silvia A. Navarro[1], Marisa Manzano[2], Priya Vizzini[2] and Natalio Krasnogor[1, *]

*natalio.krasnogor@newcastle.ac.uk, emanuela.torelli@newcastle.ac.uk

List S1. dsDNA gBlock sequences and related primers, ssRNA split sequences, ssDNA CP3, PE and PR sequences (5'-3'; Split Broccoli sequences are underlined in green).

gBlock Broccoli

TTCTAATACGACTCACTATAGGTATGTGGGAGACGGTCGGGTCCAGATATTCGTATCTGTC GAGTAGAGTGTGGGCTCCCACATACTAGCATAACCCCTTGGGGCCTCTAAACGGGTCTT GAGGGGTTTTTG

Forward primer gBlock Broccoli

TTCTAATACGACTCACTATAGGTATGTGGG

gBlock Split1d

TTCTAATACGACTCACTATAGGTCCAAAAGAAGGAACCGTATGTGGGAGACGGTCGGGT CCAGATATAGCATAACCCCTTGGGGCCTCTAAACGGGTCTTGAGGGGTTTTTG

Forward primer gBlock Split1d

TTCTAATACGACTCACTATAGGTCCAAAAGAAGGA

gBlock Split2d

TTCTAATACGACTCACTATAGGTATCTGTCGAGTAGAGTGTGGGCTCCCACATACTGGAC GAAACTTCCCCTAGCATAACCCCTTGGGGCCTCTAAACGGGTCTTGAGGGGTTTTTG

Forward primer gBlock Split2d

TTCTAATACGACTCACTATAGGTATCTGTCGAGT

gBlock complementary target

TTCTAATACGACTCACTATAGGGGAAGTTTCGTCCAGGTTCCTTCTTTTGGATAGCATAAC CCCTTGGGGCCTCTAAACGGGTCTTGAGGGGTTTTTG

Forward primer gBlock complementary target

TTCTAATACGACTCACTATAGGGGAAGTTTCG

Reverse primer

CAAAAAACCCCTCAAGACCCGTTTAG

Split1-8nt

UAGUGGCGCACGGGUGAGGUAUGUGGGAGACGGUCGGGUCCAGAUA

Split2-8nt

UAUCUGUCGAGUAGAGUGUGGGCUCCCACAUACUAAGGUAUAGUUAAUCUG

Split1-4nt

UAGUGGCGCACGGGUGAGGUGGGAGACGGUCGGGUCCAGAUA

Split2-4nt

UAUCUGUCGAGUAGAGUGUGGGCUCCCACUAAGGUAUAGUUAAUCUG

Split1-0nt

UAGUGGCGCACGGGUGAGGAGACGGUCGGGUCCAGAUA

Split2-0nt

UAUCUGUCGAGUAGAGUGUGGGCUCUAAGGUAUAGUUAAUCUG

***Campylobacter* P3 target sequence (CP3)**

CAGATTAACTATACCTTACTCACCCGTGCGCCACTA

Detection *Campylobacter* P3 sequence (CampyP3)

[biotin] TAGTGGCGCACGGGTGAGTAAGGTATAGTTAATCTG

***E. coli* sequence EF527445.1 (PE)**

AAG ACG CGC ATC TCT TTT TTC ACC AGC GCG CTT TTC

Random sequence (PR)

CGT GCG CCA CTA CAG ATT ACC TTA AAC TAT CTC ACC

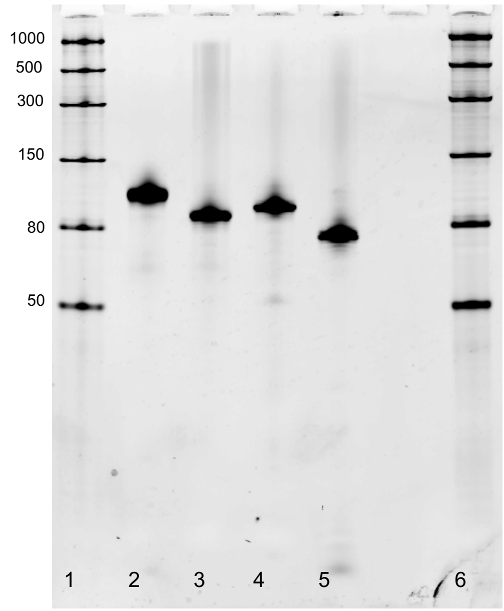

Figure S1. Broccoli aptamer and three-way junction sequences transcribed from double-stranded gBlock templates containing T7 promoter and T7 terminator. 10% TBE-Urea gel electrophoresis after SYBR® Gold staining. Lanes. 1, 6: NEB low range ssRNA ladder; 2: Broccoli aptamer (113 nt); 3: Split1d (93 nt), 4: Split2d (98 nt); 5: complementary sequence (79 nt). All the sequences include the T7 terminator (47 nt). Molecular size in nucleotides are indicated.

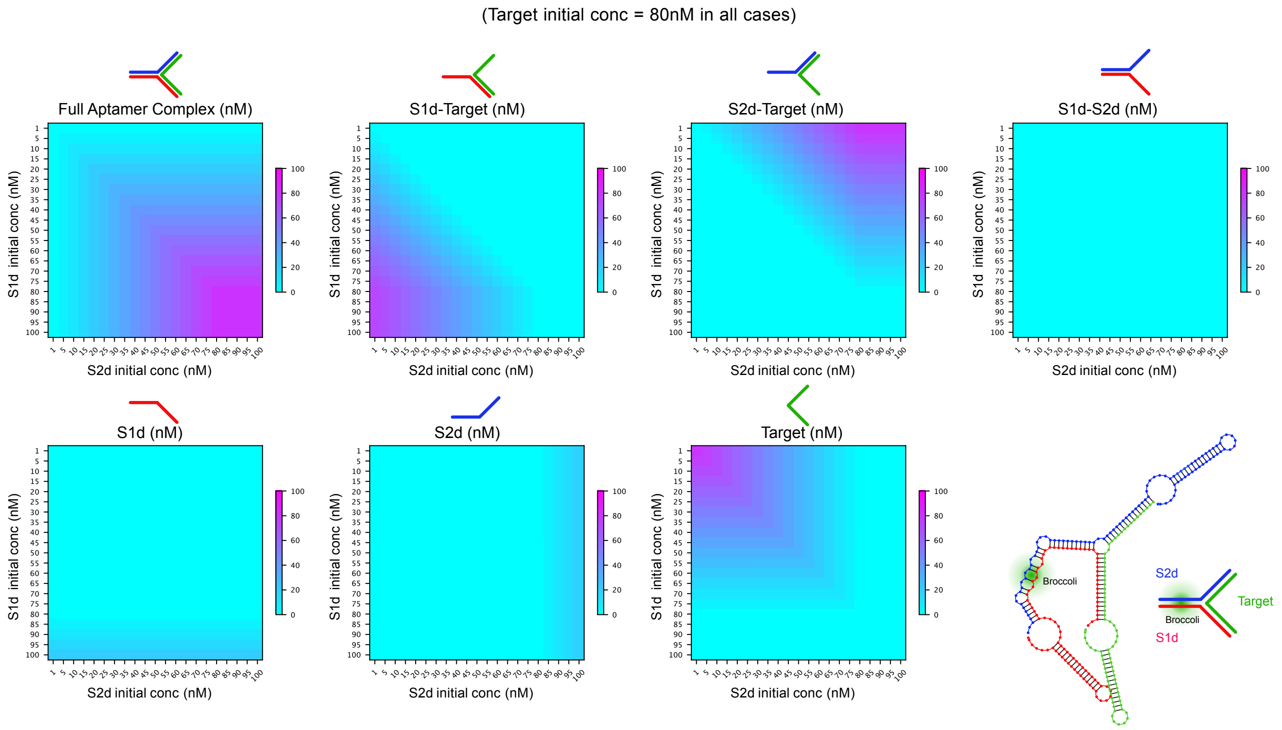

Figure S2. Computational prediction of Broccoli 3-way junction complex formation under different *in vitro* concentration conditions. The minimum free energy (MFE) structure of the full Broccoli aptamer complex is shown in bottom right. The MFE state is predicted to contain the Broccoli aptamer region, even when T7 terminator sequences feature at the 3' end of all RNA strands. Heatmaps show the predicted equilibrium concentrations of the full Broccoli aptamer complex and other partial complexes as the initial concentrations of Split1d, Split2d are varied. The concentration of the target strand is constant at 80 nM in all plots. The heatmap's results apply to the non/T7 terminator case and also to the T7 terminator case. From the heatmaps it can be observed that: (i) Split1d and Split2d are predicted not to hybridise without target (ii) Split1d-target complexes exist when Split2d is at relatively low concentration compared to Split1d and target (iii) Split2d-target complexes exist when Split1d is at relatively low concentration compared to Split2d and target (iv) The full 3-strand Broccoli complex is reliably formed when the concentrations of Split1d and Split2d are equal to, or greater than, the concentration of target. Figure created with NUPACK v4.0.0.27 test tube analysis. Predictions are considered at 37 °C in 1 M Na^+ and 0 M Mg^{2+}.

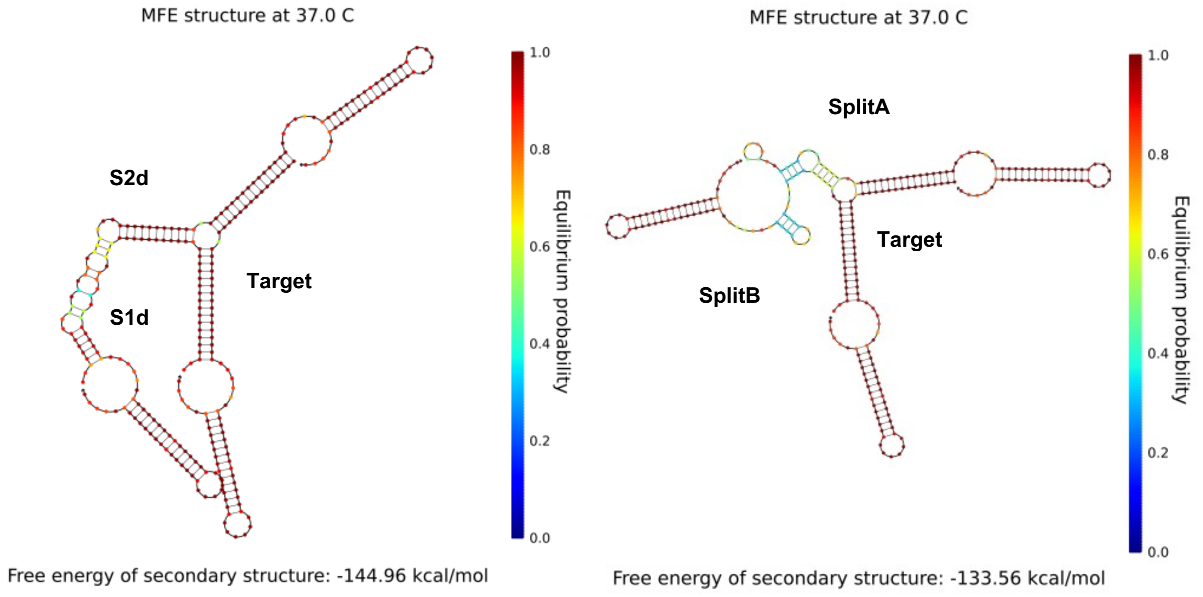

Figure S3. NUPACK secondary structure prediction of Split1d, Split2d and target full complex (left), and of SplitA, SplitB and target full complex (right). The sequences' concentrations are 120 nM at 37 °C in 1 M Na^{+} and 0 M Mg^{2+}.

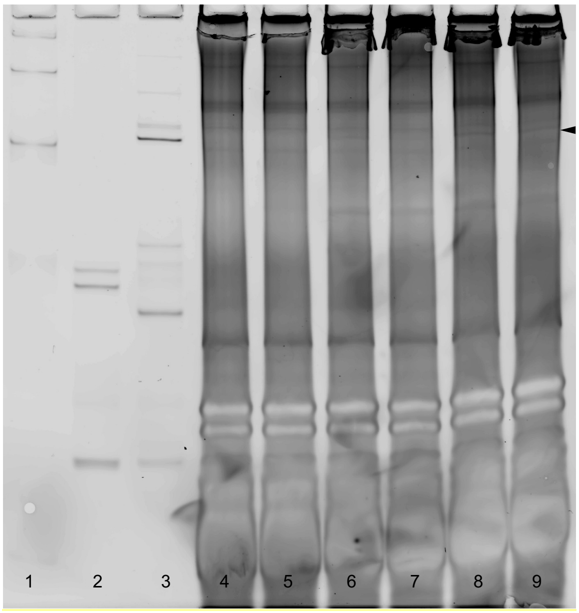

Figure S4. PURExpress cell-free system containing the three-way junction gBlock templates analysed by polyacrylamide gel. 10% TBE gel electrophoresis after SYBR® Gold staining. Lanes: 1: low range ssRNA ladder NEB; 2: Split1d and Split2d (after incubation at 37°C for 25 min); 3: Split1d, Split2d and target (after incubation at 37°C for 25 min); 4: cell-free system; 5: gBlock Split1d and Split2d in cell-free system; 6, 7: gBlock Split1d, Split2d and target in cell-free system loading 2.5 µL; 8, 9: gBlock Split1d, Split2d and target in cell-free system loading 3.5 µL. Cell-free system samples are loaded after incubation at 37 °C and fluorescence measurements using qPCR thermal cycler. The black arrow underlines the 3-way junction assembled in the cell-free system.

List S2. de Bruijn scaffold sequence to be transcribed (inserted into the plasmid pUCIDT-AMP-T7p_DB982; in red T7 promoter sequence, in black the scaffold sequence to be transcribed) and primers sequences (5'-3'). The dsDNA is amplified using T7-DB982 forward and T7-DB982 reverse primers and purified. The purified dsDNA template is then transcribed in RNA scaffold by T7 polymerase.

TAATACGACTCACTATAGGCGGTCCAGCTAGCAGGTTTGCGGCTCAGAAGAGCTGTTGT
GTTTGTTTTCGACTACCAGAACGGAGTCTCTAGCGTGAGATAAGTAAGATTAGGCTCGG
AGAGTGTGAGGCTTCGTAATCGTACCACACACCAGGCGTAACCGCACTTAGACGCACAG
GGTACAAGTGATAGGTAAAGTTACGGCAGGACGCCCAAAAGTCTGGAGCACAAACGGG
GCCCCGCTAGGGAAAACGCCGGGGTAACTATTGTTATAATTCAAGAATTAGAACTAAAA
GGTAGTAGCACCACTCGGTGGGTTAAACTAGCTAAAGACACCGCTCCAACAGCCGAAA
GTGTACGCTGAATCACAGTCAAATTATACGGTGTTCGAGATCGCGAGTTTTGTGGGATTT
GCACTCCAGATACCGATTCGGTAGCTTTATCGTTCACTGTGTCACGCGCAGCGCCACCAA
AGCTGAGACGTTCTCGAAATTCTAATTTCTACGATTAAGTCCAAACAGAAAGCAATCTAT
TACACTGGAAGTCAGTAAAACAAAGGGATACAGATCCCGTGACGGCTAGTGCTGTGGTG
TCCGAAGTTGACTGTCAGAGAACAATCGCACCGGACAGTTCGTTGAGTTCCAGTTGCA
ATTGCGATAGTAATAATAGATAGAGGCCGTGGAACCCCGTACTTCAGCGAGAAGTGGTCT
TGGACTTGTACTGGGGCGAGCGGTGCGGGAACTCGTGTTGCCCGCAAGCACTGCAACA
CAGCGGAAGGATAGCAACGATCACTCTTGCTTTGTCGGACTCAGTCTAGGAGCCGCCGA
GCCAGTCCCGCGCGTTCCCACGTTTCCGTAAACGTCCGCTTGGCCCGTCCACTGATATAG
TTGGATCGGGAGAAATCGAAGCTCACGAACAGGAACGTAAGGCTGCTTGTTCTTTCACG
GATCTCGGGCAGAATCTCAAACTCAATTACTCGATTTAGGTCGTCGCAGTACAG

Primer T7-DB982 Forward

TAATACGACTCACTATAGGCGGTCC

Primer T7-DB982 Reverse

CTGTACTGCGACGACCTAAATCG

Table S1. Single-stranded DNA and RNA staple strands sequences (in green underlines the split Broccoli system).

Start	End	Sequence (5'-3')	Length
4[95]	3[95]	ACTAGCCGTCACGGGAACAGTGAACGATAAAG	32
4[79]	5[79]	TCTGTATCCCTTTGTTTCTCGCCCCAGTACAAGT	34
2[111]	3[111]	AGCGGTGTCTTTAGCTACTACCGAATCGGTATCT	34
7[112]	7[127]	AGAACAAGCAGCCTTA	16
2[47]	3[47]	TATAACAATAGTTACCCAGAAATTAGAATTTCGA	34
0[143]	1[135]	CTAATCTTACTTATCTCGTACGATT	25
4[47]	5[47]	AATAGATTGCTTTCTGTCAGTGCTTGCGGCAAC	34
6[111]	7[111]	GTTTACGGAAACGTGGGTGCCCGAGATCCGTGAA	34
5[136]	7[143]	CGCAATTGCATCCAACTATATCAGTGCGTTCCTGTTCGTGAGC	43
6[79]	7[79]	TCGGCGGCTCCTAGACTACGACCTAAATCGAGTA	34
2[63]	1[63]	GTTCTAATTCTTGAATCTCCAGACTTTTGGGC	32
2[95]	1[95]	GTTTAACCCACCGAGTCCTATCACTTGTACCC	32
2[95]	1[95]	GUUUAACCCACCGAGUCCUAUCACUUGUACCCGGGAGACGGUCGGGUCCAGAUA	56
6[63]	5[63]	GAGTCCGACAAAGCAAACGAGTTCCCGCACCG	32
2[79]	3[79]	GGTGCTACTACCTTTTAGTGGCGCTGCGCGTGAC	34
6[95]	5[95]	AACGCGCGGGACTGGCCCAAGACCACTTCTCG	32
0[47]	1[47]	TCACAGAACCGTGCGCCGCGGGGCCCCGTTTGTG	34
4[111]	5[111]	ACTTCGGACACCACAGCCTGAAGTACGGGGTTCC	34
0[79]	1[79]	TGAGCCGCAAACCTGCTGTCCTGCCGTAACTTTA	34
3[136]	5[135]	CGATCTCGAGAACTGTCCGGTGCGATATTACTAT	34
0[95]	0[80]	AACACAACAGCTCTTC	16
0[95]	0[80]	UAUCUGUCGAGUAGAGUGUGGGCUCCCAACACAACAGCUCUUC	43
6[127]	5[127]	GACGGGCCAAGCGGACACGGCCTCTATCTATT	32
0[63]	0[48]	AGCTGGACCGCCACGA	16
4[63]	3[63]	TACTGACTTCCAGTGTGAACGTCTCAGCTTTG	32
0[111]	1[111]	TCTGGTAGTCGAAAACATGTGCGTCTAAGTGCGG	34
6[47]	7[47]	GAGTGATCGTTGCTATCGAGCTATTTCAAGCCCG	34
1[136]	3[135]	ACGAAGCCTGACTGTGATTCAGCGTAAAAACTCG	34
4[127]	3[127]	TGTTCTCTGACAGTCAGGAGTGCAAATCCCAC	32
7[80]	7[95]	ATTGAGTTTGAGATTC	16
2[127]	1[127]	CACTTTCGGCTGTTGGTTACGCCTGGTGTGTG	32
0[127]	0[112]	ACGCTAGAGACTCCGT	16
7[48]	7[63]	TGGAAGCTGTACTGCG	16

Table S2. Bacterial sequences considered for the probe specificity test.

Bacteria	16S and 16S-23S sequences	Whole DNA sequence
Campylobacter fetus	KP064555.1	
C. concisus	GQ167709.1	
C. gracilis	JX912515.1	
C. concisus	L04322.1	
C. fetus subsp. fetus	AB301966.1	
C. fetus subsp. venerealis	M65011.1	
C. hyointestinalis	NZ_FPBB01000005.1	
C. mucosalis	NZ_jhqq01000009.1	
C. sputorum biovar sputorum	NZ_jmti01000045.1	
Aeromonas sobria	EF373994.1	kv861263.1
Bacillus cereus	AM062666.1	ap007209.1
B. subtilis	EF205020.1	NZ_cp011534.1
Citrobacter freundii	AF047423.1	CP016762.1
Escherichia coli	EF527445.1	ba000007.2
Enterobacter aerogenes	FJ410387.1	NZ_LYDO01000004.1
E. cloacae	EU078570.1	NZ_cp011798.1
Helicobacter ganmani	AY277975.1	
Helicobacter pylori b38		fm991728.1
Klebsiella pneumoniae	DQ399570.1	fo203501.1
Lactobacillus plantarum	AB092638.1	NZ_cp012650.1
Legionella pneumophila	AF000655.1	NZ_cp011105.1
Leuconostoc lactis	AB295116.1	
Listeria monocytogenes	AY684791.1	NZ_bbrp01000009.1
Morganella morganii	AB089244.1	
Pediococcus pentosaceus	AF405374.1	
Proteus vulgaris	FJ518598.1	
Pseudomonas aeruginosa	JN418884.1	
Ps. brennerii	AM086254.1	lvwz01000001.1
Ps. fluorescens	EF198908.1	NZ_LDET01000015.1
Salmonella enterica	AF046822.1	NC_003197.2
Shigella sonnei	KX696458.1	NZ_lyem01000481.1
Staphylococcus aureus	U11784.1	
Vibrio parahaemolyticus	AY531067.1	NZ_lirr01000034.1
Weissella cibaria	FJ429988.1	NZ_cp012873.1
Yersinia enterocolitica	AF293850.1	am286415.1

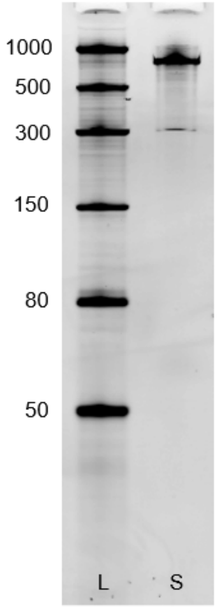

Figure S5. Transcribed and purified de Bruijn scaffold sequence (DBS982) analysed by denaturing polyacrylamide gel. 10% TBE-Urea gel electrophoresis after SYBR® Gold staining. Lanes. L: NEB low range ssRNA ladder; S: de Bruijn scaffold sequence (DBS982). Molecular sizes in nucleotides are indicated.

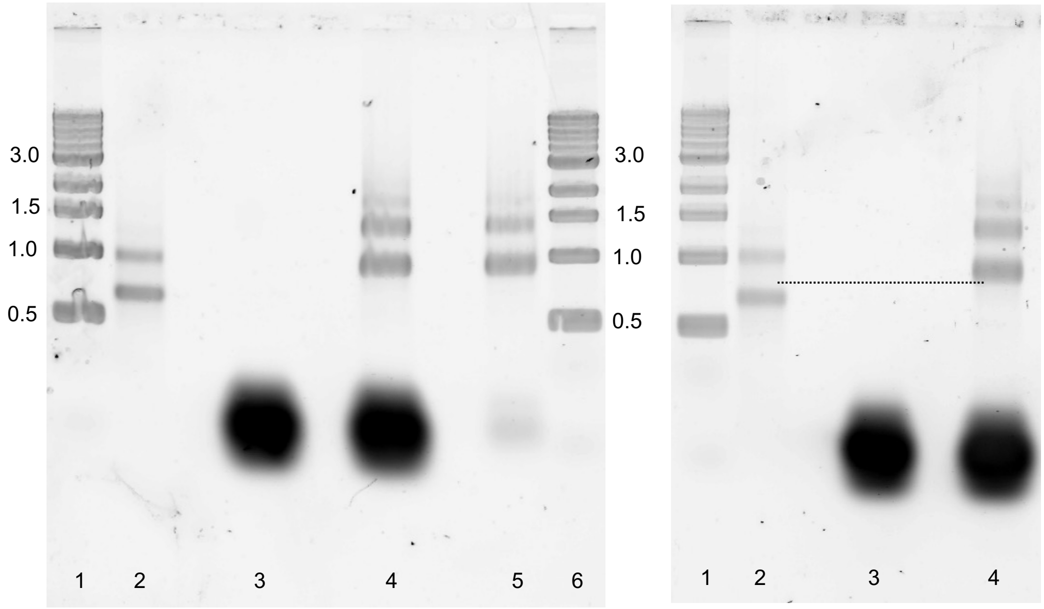

Figure S6. Scaffold, staple strands, unpurified and purified hybrid RNA/DNA origami analysed by agarose gel. 1.5% TAE agarose gel electrophoresis after SYBR® Gold staining. Lanes. 1 and 6: 1 kb DNA ladder; 2: transcribed and purified DBS982 RNA scaffold; 3: staple strands; 4: hybrid RNA/DNA origami; 5: hybrid RNA/DNA origami purified using Amicon Ultra centrifugal filter. Molecular sizes in kilobases are indicated.

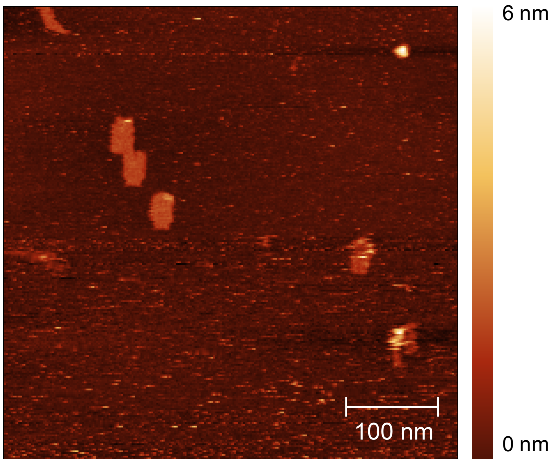

Figure S7. High-resolution AFM image of hybrid rectangle RNA/DNA origami.

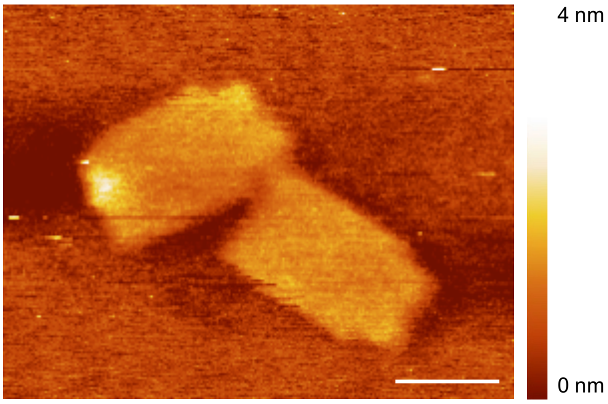

Figure S8. High-resolution AFM image of hybrid rectangle DNA-RNA origami (scale bar 20 nm).

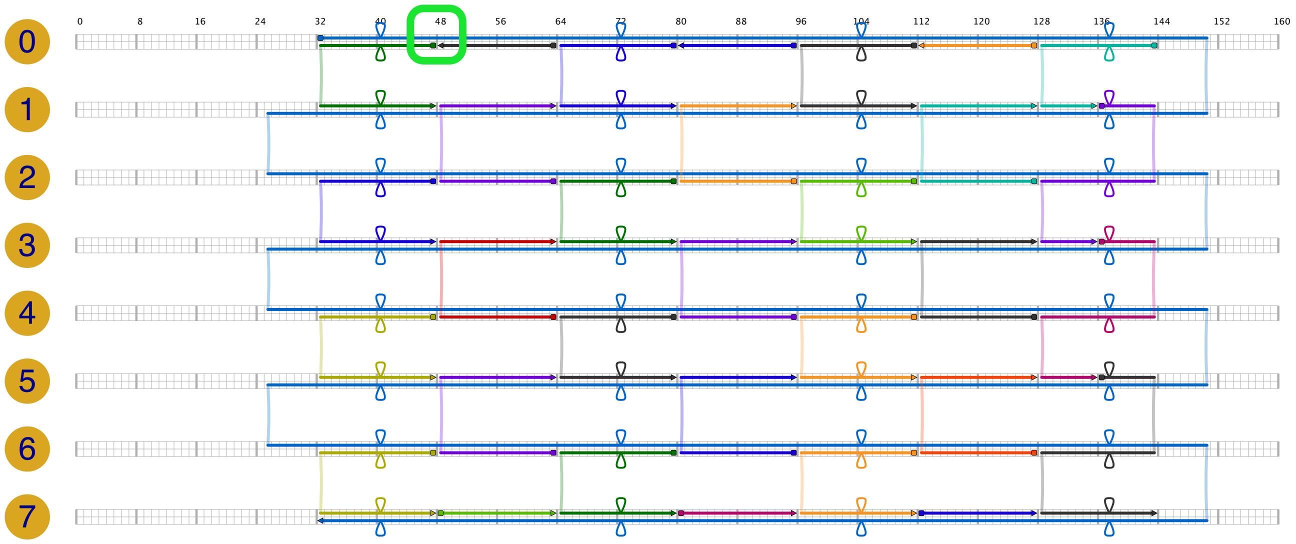

Figure S9. Scadnano schematic design of the hybrid RNA/DNA origami. The green box underlines the Split system position. The scaffold is underlined in blue, staple strands are shown in different colours.

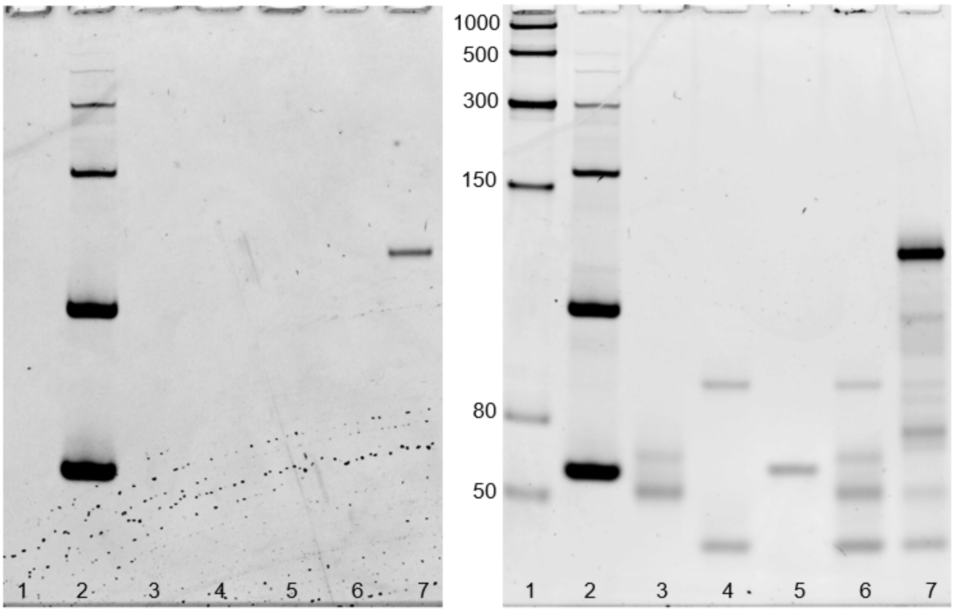

Figure S10. In-gel imaging of staple S1 and staple S2 hybridized with complementary DBS sequence. 10% TBE polyacrylamide gel after DFHBI-1T (left) and after SYBR® Gold (right)

staining. The gel was stained with DFHBI-1T to visualize Broccoli aptamer (positive control), and the hybridized staple S1, staple S2 and complementary DBS target. After 3 washing steps, the gel was stained with SYBR® Gold for 5 min. Lanes: 1: low range ssRNA ladder; 2: Broccoli aptamer; 3: modified staple S1; 4: modified staple S2; 5: complementary DBS target sequence; 6: modified staple S1 and S2; 7: modified staple S1 and S2, and complementary DBS target sequence. Molecular sizes in nucleotides are indicated.

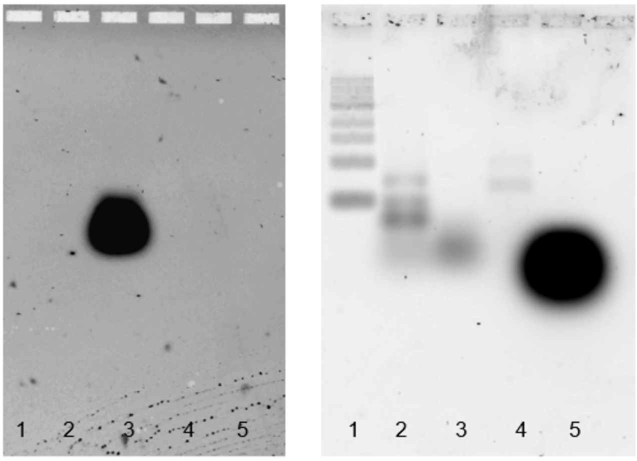

Figure S11. In-gel imaging of Broccoli aptamer, RNA scaffold mix and staple strands mix. 1.5% TAE agarose gel after DFHBI-1T (left) and after SYBR® Gold (right) staining. The gel was stained with DFHBI-1; after 3 washing steps, the gel was stained with SYBR® Gold for 10 min. Lanes: 1: 1 Kb ladder; 2: low range ssRNA ladder; 3: Broccoli aptamer; 4: RNA scaffold; 5: staple strands.

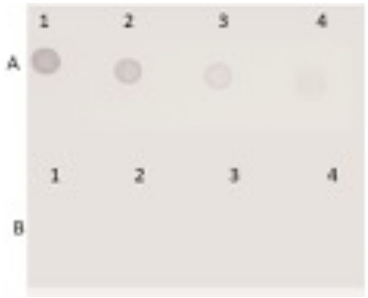

Figure S12. Dot blot detection of biotin-labelled CampyP3 detection probe using different dilution of target complementary CP3 sequence. Row A. A1: 100 ng/µL, A2: 50 ng/µL, A3: 10 ng/µL, A4: 1 ng/µL. **Row B.** B1: 0.1 ng/µL, B2: 0.01 ng/µL, B3: 0.001 ng/µL, B4: 0.0001 ng/µL.

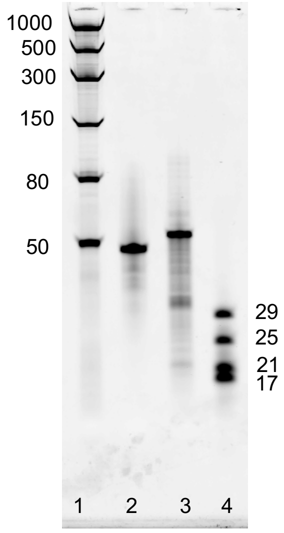

Figure S13. Split1-8nt and Split2-8 nt analysed by denaturing polyacrylamide gel. 10% TBE-Urea polyacrylamide gel after SYBR® Gold staining. Lanes. 1: NEB low range ssRNA ladder; 2: Split1-8nt (46 nt), 3: Split2-8nt (51 nt); 4: ZR small-RNA™ ladder. Molecular sizes in nucleotides are indicated.

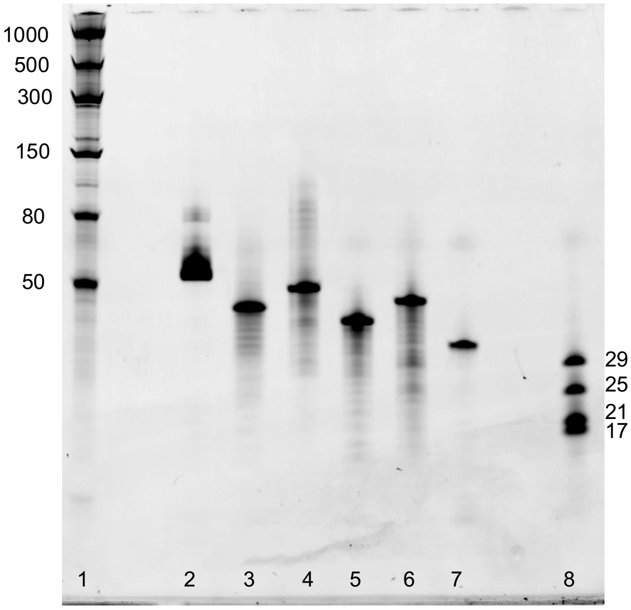

Figure S14. Broccoli aptamer, Split1-4nt, Split2-4nt, Split1-0nt, Split2-0nt and CP3 sequences analysed by denaturing polyacrylamide gel. 10% TBE-Urea polyacrylamide gel after SYBR® Gold staining. Lanes. 1: NEB low range ssRNA ladder; 2: Broccoli aptamer (66 nt); 3: Split1-4nt (42 nt), 4: Split2-4nt (47 nt); 5: Split1-0nt (38 nt); 6: Split2-0nt (43 nt); 7: ssDNA CP3 target; 8: ZR small-RNA™ ladder. Molecular sizes in nucleotides are indicated.

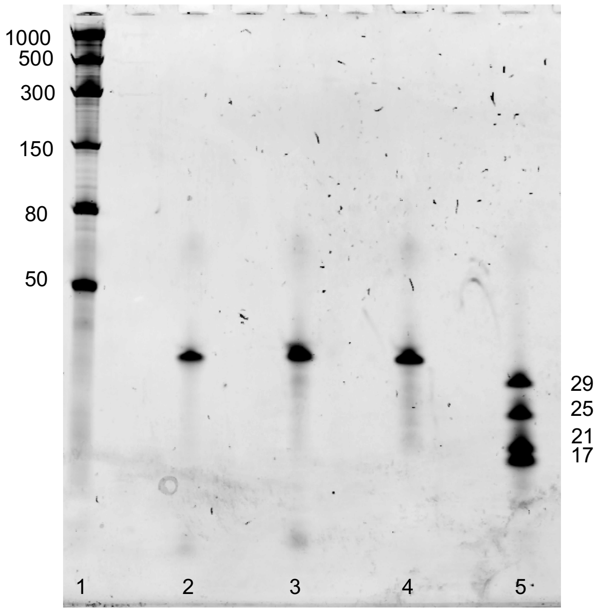

Figure S15. CP3, PE and PR sequences analysed by denaturing polyacrylamide gel. 10% TBE-Urea polyacrylamide gel after SYBR® Gold staining. Lanes. 1: low range ssRNA ladder; 2: ssDNA target CP3; 3: ssDNA PE (negative control); 4: ssDNA PR (negative control); 5: ZR small-RNA^{TM} ladder. Molecular sizes in nucleotides are indicated.

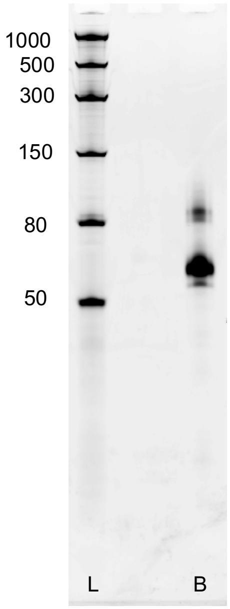

Figure S16. Transcribed and purified broccoli sequence analysed by denaturing polyacrylamide gel. 10% TBE-Urea polyacrylamide gel after SYBR® Gold staining. Lanes. L: low range ssRNA ladder; B: Broccoli aptamer. Molecular sizes in nucleotides are indicated.

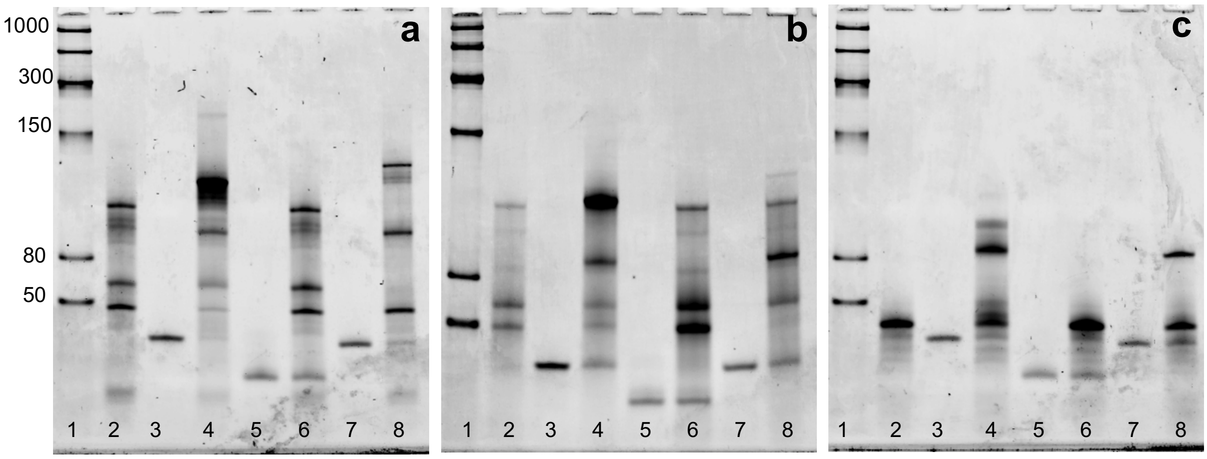

Figure S17. Comparison of hybridization reactions in 20 mM Tris-HCl pH 7.5, 1 mM EDTA and 10 mM $MgCl_2$, using Split1/Split2-8nt, Split1/Split2-4nt or Split1/Split2-0nt and CP3, PE or PR sequences. 10% TBE polyacrylamide gel after SYBR® Gold staining. **a**: Split1/Split2-8nt; **b**: Split1/Split2-4nt; **c**: Split1/Split2-0nt. Lanes: 1: low range ssRNA ladder; 2: Split1 and Split2; 3: target CP3; 4: Split1/Split2 and target CP3; 5: PE; 6: Split1/Split2 and PE (negative control); 7: PR (negative control); 8: Split1/Split2 and PR. Molecular sizes in nucleotides are indicated.

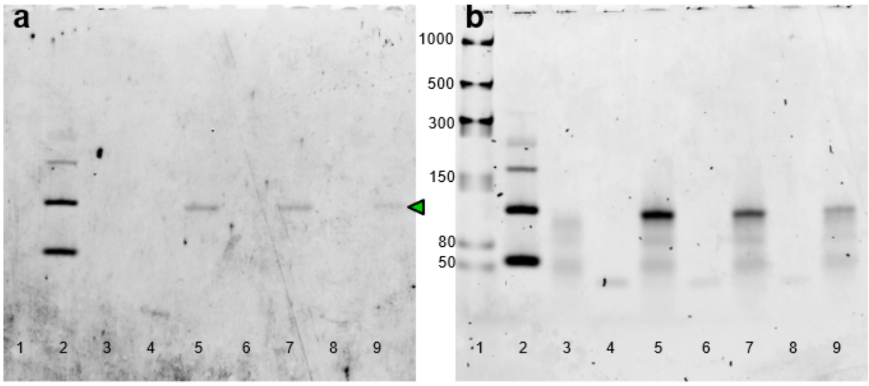

Figure S18. In-gel imaging of the split system in the presence of CP3 target sequences at different concentrations. 6% TBE polyacrylamide gel after DFHBI-1T (a) and after SYBR® Gold (b) staining. The gel was stained with DFHBI-1T for 4 min to visualize Broccoli aptamer (positive

control), and the hybridized Split1/Split2-4nt/CP3. After 3 washing steps, the gel was stained with SYBR® Gold for 5 min. Lanes: 1: low range ssRNA ladder; 2: Broccoli aptamer; 3: Split1/Split2-4nt (0.12 μM); 4: target CP3 (0.10 μM, 1.2 ng/μL); 5: Split1/Split2-4nt (0.12 μM each) and target CP3 (0.10 μM, 1.2 ng/μL); 6: target CP3 (0.08 μM, 0.9 ng/μL); 7: Split1/Split2-4nt (0.12 μM each) and target CP3 (0.08 μM, 0.9 ng/μL); 8: target CP3 (0.05 μM, 0.6 ng/μL); 9: Split1/Split2-4nt (0.12 μM each) and target CP3 (0.05 μM, 0.6 ng/μL). Molecular sizes in nucleotides are indicated; the green arrow underlines the reconstituted split Broccoli aptamer.

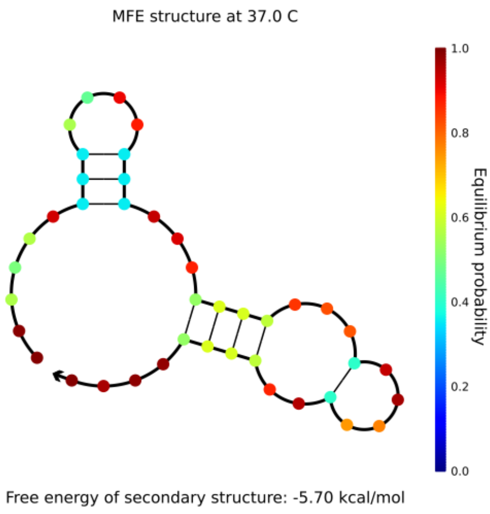

Figure S19. NUPACK secondary structure prediction of Split1-4nt sequence (50 nM) at 37 °C in 1 M Na^+ and 0 M Mg^{2+}.

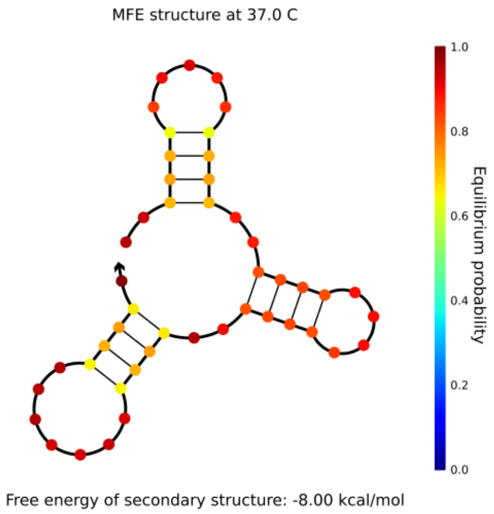

Figure S20. NUPACK secondary structure prediction of Split2-4nt sequence (50 nM) at 37 °C in 1 M Na^+ and 0 M Mg^{2+}.

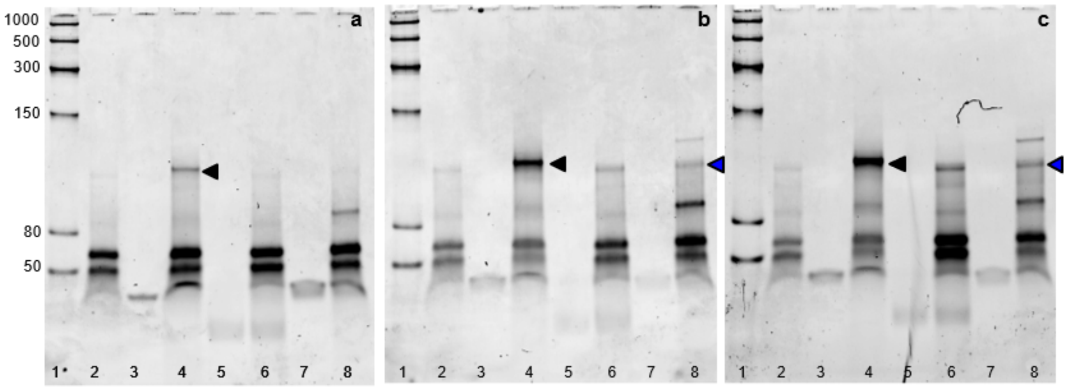

Figure S21. Comparison of hybridization reactions using Split1/Split2-4nt and CP3, PE or PR sequences in DFHBI-1T aptamer buffer (40 mM HEPES pH 7.4, 100 mM KCl, 1 mM $MgCl_2$) without or with different $MgCl_2$ concentrations. Samples are run in 10% pre-cast Novex^{TM} TBE gel in 1x TBE buffer at 200 V for 40 min and stained SYBR® Gold. Hybridization reactions using Split1/Split2-4nt are performed in: a) DFHBI-1T aptamer buffer; b) DFHBI-1T aptamer buffer supplemented with 5 mM $MgCl_2$; c) DFHBI-1T aptamer buffer supplemented with 10 mM $MgCl_2$. Lanes: 1: low range ssRNA ladder; 2: Split1-4nt and Split2-4nt; 3: target CP3; 4: Split1/Split2-4nt

and target CP3; 5: PE; 6: Split1/Split2-4nt and PE (negative control); 7: PR (negative control); 8: Split1/Split2-4nt and PR. Molecular sizes in nucleotides are indicated. Black arrows: Split1/Split2-4nt/CP3 hybrid; blue arrows: low partial hybridization between Split1-4nt and Split2-4nt. The high aptamer buffer salt concentration can result in an increased conductivity that can affect migration and result in bent lower bands (gel artefacts), not visible when the hybridization buffer (20 mM Tris HCl pH 7.6, 1 mM EDTA, 10 mM $MgCl_2$, Figure S22) is used.

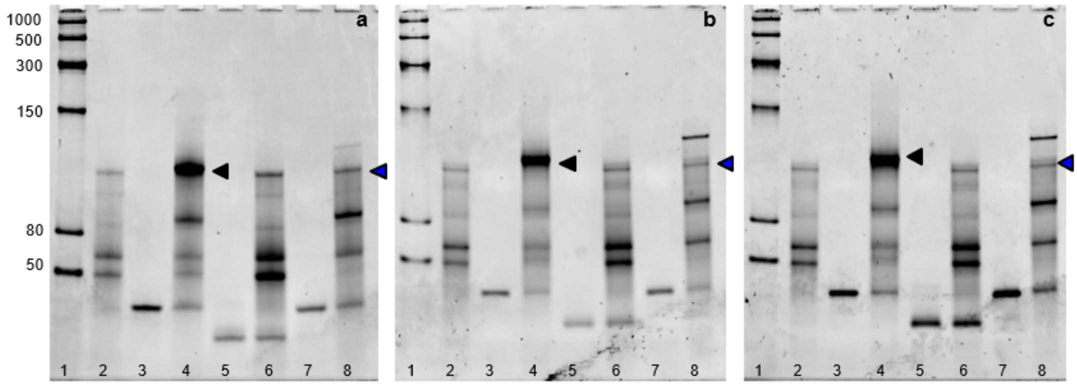

Figure S22. Comparison of hybridization reactions using Split1/Split2-4nt and CP3, PE or PR sequences in hybridization buffer without or with different KCl concentrations. Samples are run in 10% pre-cast Novex^{TM} TBE gel in 1x TBE buffer at 200 V for 40 min and stained SYBR® Gold. Hybridization reactions are performed in: a) 20 mM Tris HCl pH 7.6, 1 mM EDTA, 10 mM $MgCl_2$ (hybridization buffer); b) 20 mM Tris HCl pH 7.6, 1 mM EDTA, 10 mM $MgCl_2$ supplemented with 50 mM KCl; c) 20 mM Tris HCl pH 7.6, 1 mM EDTA, 10 mM $MgCl_2$ supplemented with 100 mM KCl. Lanes: 1: low range ssRNA ladder; 2: Split1-4nt and Split2-4nt; 3: target CP3; 4: Split1/Split2-4nt and target CP3; 5: PE; 6: Split1/Split2-4nt and PE (negative control); 7: PR (negative control); 8: Split1/Split2-4nt and PR. Molecular sizes in nucleotides are indicated. Black arrows: Split1/Split2-4nt/CP3 hybrid; blue arrows: low partial hybridization between Split1-4nt and Split2-4nt.
